# Evidences for Piperine inhibiting cancer by targeting human G-quadruplex DNA sequences

**DOI:** 10.1038/srep39239

**Published:** 2016-12-20

**Authors:** Arpita Tawani, Ayeman Amanullah, Amit Mishra, Amit Kumar

**Affiliations:** 1 Centre for Biosciences and Biomedical Engineering, Indian Institute of Technology Indore, Indore, Madhya Pradesh, 453552, India; 2 Cellular and Molecular Neurobiology Unit, Indian Institute of Technology Jodhpur, Rajasthan, 342011, India

## Abstract

Piperine, a naturally occurring alkaloid, is well known as anti-oxidant, anti-mutagenic, anti-tumor and anti-proliferative agent. Piperine exerts such pharmacological activities by binding or interacting with various cellular targets. Recently, the first report for Piperine interaction with duplex DNA has been published last year but its interaction with G-quadruplex structures has not been studied yet. Herein, we report for the first time the interaction of Piperine with various DNA G-quadruplex structures. Comprehensive biophysical techniques were employed to determine the basis of interaction for the complex formed between Piperine and G-quadruplex DNA sequences. Piperine showed specificity for G-quadruplex DNA over double stranded DNA, with highest affinity for G-quadruplex structure formed at c-myc promoter region. Further, *in-vitro* studies show that Piperine causes apoptosis-mediated cell death that further emphasizes the potential of this natural product, Piperine, as a promising candidate for targeting G-quadruplex structure and thus, acts as a potent anti-cancer agent.

G-quadruplex DNA structures are classified as non-canonical DNA structures that were formed by square planar arrangement of G-quartets. Apart from Watson-crick hydrogen bonding, these G-quartets are stabilized by Hoogsteen hydrogen bonding^1^. This non-canonical form of DNA was formed by guanine-rich sequences and is widespread in human genome. Approximately, 300,000 sequences have ability to form G-quadruplex structures^2^ and majority of them includes telomeres^3^, regulatory regions of oncogenes such as c-kit, c-myc^4^, and hence makes these regions as a potent pharmacological targets for anti-tumor or anti-cancer therapeutics. Telomeric DNA contains repetitive DNA sequence (TTAGGG)_n_ forming G-quadruplex structures; this structures inhibits telomerase activity that is required to maintain telomeres^5^. As in 85% of cancers, the activity of this enzyme has been found to be elevated, thus, inhibition of its activity could be an striking approach in the advancement of anticancer drugs development^6^. Another impressive target is G-quadruplex structure formed at promoter region of c-myc gene. This c-myc proto-oncogene regulates approximately 15% of all gene expression and controls various processes of cell-cycle regulation such as apoptosis, growth and proliferation. Its overexpression has been found to be associated with sustained tumor progression. The promoter region of c-myc gene is composed of seven nuclease-hypersensitive elements (NHEs), of which, NHE III_1_, located at −142 to −115 base pairs upstream of the P1 promoter, controls 80–90% transcription of c-myc gene^7^,^8^. This 27 nucleotide sequence (5′-TGGGGAGGGTGGGGAGGGTGGGGAAGG-3′) is purine rich sequence which is also called as Pu27, has potential to form G-quadruplex structure^9^,^10^. It has been known that this NHE III_1_ element could form transcriptionally active and silenced forms (single stranded and duplex DNA respectively). The transcriptional silencing of this promoter is believed to be achieved by the formation of G-quadruplex DNA structures. This was also evident from one of the studies in which suppression of MYC expression was observed when Burkitt lymphoma cell lines was treated with TMPyP4 aids in the formation of stable G-quadruplex structure^11^. Ligands have been reported to interfere with transcription of c-myc gene by stabilizing G-quadruplex structure^11^,^12^,^13^,^14^.

Moreover in last few years various ligands with synthetic and natural origin have been reported that binds to various human G-quadruplex DNA like telomeric DNA^15^, promoter region of c-myc DNA^16^. One of such naturally available small molecule is Piperine. It is a chief alkaloid from black pepper (*Piper nigrum* L.) and from the times of Ayurveda, this phytochemical is known for its various pharmacological and physiological properties^17^. These activities include antifungal, antimicrobial^18^, antidepressant^19^, antipyretic^20^, anti-oxidant^21^, anti-inflammatory, anti-apoptotic^22^, etc. It has also been reported that Piperine enhances the bioavailability of other phytochemicals^23^ and drugs, for example, rifampcin^24^, resveratrol^25^, etc. This naturally available non-toxic molecule has been used for the treatment of leukemia, malaria^26^ as well leshmianasis^27^. Piperine also inhibits Akt phosphorylation^28^ and suppresses angiogenesis as well as it exerts its anti-cancer effect by inhibiting CREB, NF-kB, c-Fos activities^29^. The beneficial effect of Piperine towards human health makes it a suitable candidate for targeting macromolecules inside cells. Despite of these studies, the molecular mechanism for action of Piperine with biologically significant macromolecules is not fully studied yet. In literature, few reports are available for the interaction of Piperine with proteins such as bovine β-lactoglobulin^30^, chicken α1-acid glycoprotein^31^ and human serum albumin^32^. However, DNA is often a potential target for antibiotic, anti-fungal, anti-viral, anti-tumor and anti-cancer drugs. Very recently, Haris *et al*. has reported the interaction of Piperine with duplex DNA^33^. This study revealed the molecular mechanism of interaction of Piperine with calf thymus DNA and showed that Piperine binds in minor groove of DNA. Howbeit in anti-cancer drug discovery domain, structure specific targeting of drugs provides an aid for improving drug specificity and affinity for specific targets. Structures formed by G-quadruplex DNA are potent targets for anti-cancer drug discovery^34^. Small molecule ligands with high specificity and affinity for G-quadruplex structures could be used as potent therapeutics for targeting cancer by regulating the gene expression. Generally, molecules with planar and aromatic ring system provide scaffolds that assists in the binding and stabilizing G-quadruplex structure by π- π stacking^35^. Piperine has aromatic ring and has planar structure that could provide a framework for end-stacking or π- π stacking with G-quadruplex structure. Further credence has been lent to the strategy to explore Piperine because there were no reports available for its interaction with any of DNA sequences forming G-quadruplex structures.

Herein this study, we have chosen three biologically significant DNA sequence forming G-quadruplex structure viz human telomeric DNA tel22, (d-5′-AGGGTTAGGGTTAGGGTTAGGG-3′), promoter region of c-kit21 (d-5′-CGGGCGGGCGCGAGGGAGGGG-3′) and c-myc promoter G-quadruplex sequence Pu24T (5′-TGAGGGTGGTGAGGGTGGGGAAGG-3′) and studied their interaction with Piperine (Fig. 1). In order to understand this interaction, various biophysical techniques were employed such as circular dichroism (CD), DNA melting studies, steady-state and time-resolved fluorescence spectroscopy and proton NMR spectroscopy. Moreover, computational analysis for the dynamics of Piperine-Pu24T DNA interaction were also performed by using docking and molecular dynamics (MD) simulation methods. Furthermore, *in vitro* studies were employed to understand the cytotoxic effects of Piperine on various cancer cell lines, explored its mechanism of action on human lung carcinoma (A549) cell lines and established its potential to down-regulate c-myc gene expression in cancer cells.

## Results and Discussion

### Steady state and Time resolved fluorescence titration studies

**Figure 2:**
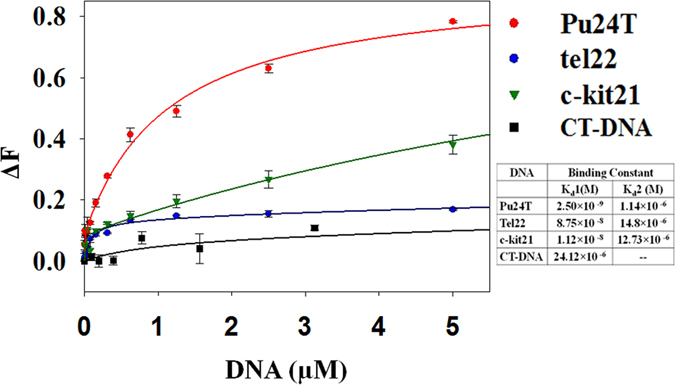
Steady state fluorescence studies. Fluorescence titration curve of Piperine as a function of various DNA concentration: Red: Pu24T, Blue: tel22, Green: c-kit21 and Black: CT-DNA. Solid lines represent fit according to the ligand binding two site saturation model and both the binding constant values(K_d_1 and K_d_2) are indicated at the bottom right side of the plot.

We first investigated the binding of Piperine to G-quadruplex DNA by employing fluorescence titration experiment and data was analysed at the emission maximum of its unbounded form. Piperine is a strong fluorophore that has emission maximum at a wavelenght of 486 nm when excited at 341 nm. With an incremental addition of DNA to Piperine solution, an increase in fluorescence intensity was observed that depicts the binding of Piperine with DNA resulting in the formation of DNA-Piperine complex. The obtained binding curve was fitted with two site saturation model of ligand binding (Fig. 2) that gives the binding constant values (k_d_1 and k_d_2) for two different affinities of Piperine for two different sites (see Supplementary Table S1). This result propounded the higher affinity of Piperine for Pu24T c-myc G-quadruplex DNA as compared to other G-quadruplex DNA used in this study. Apart from high affinity for Pu24T, Piperine showed high specificity towards G-quadruplex structure as compared to double stranded calf thymus duplex DNA with ~10^4^ fold higher affinity for G-quadruplex structures. Our result from fluorescence titration experiment suggested a specific binding of Piperine for Pu24T c-myc G-quadruplex structure with highest affinity. However, the observed differences in the affinity for various G-quadruplex DNAs could be due to difference in their sequences and topologies^36^,^37^.

**Figure 3:**
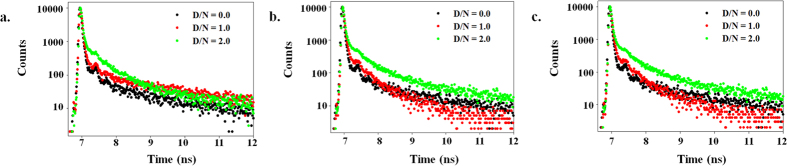
Time resolved fluorescence studies. Fluorescence life time decay curve of 40.0 μM Piperine (Black) and its complex with G-quadruplex DNA at D/N ratio = 1.0 (Red) and D/N ratio: 2.0 (Green): (**a)** Pu24T **(b**) tel22 **(c**) c-kit21.

In order to explore the environment of fluorophore in its excited state, we have measured the fluorescence lifetime decay profile of Piperine in absence and presence of various G-quadruplex DNA. This method is very sensitive for excited-state interactions between ligand and macromolecules and therefore fluorescence lifetime data could provide an understanding about the interaction behaviour of Piperine and G-quadruplex DNA. Figure 3a,b and c displayed the fluorescence decay profiles of Piperine and its complex with G-quadruplex DNA at (Drug/Nucleic Acid) D/N = 1.0 and 2.0 ratio. It is clearly evident that with addition of DNA, the life time decay profile of Piperine is increased, that might be due to changes in the environment of Piperine and thus corroborates the binding of Piperine to DNA and formation of drug-DNA complex. Further, we have also examined life time decay profile of Piperine when bound to CT-DNA (see Supplementary Fig. S1) and found that there was no significant change in it is decay profile.

### Circular Dichroism and Thermal denaturation studies for the interaction of Piperine with G-quadruplex DNA

**Figure 4:**
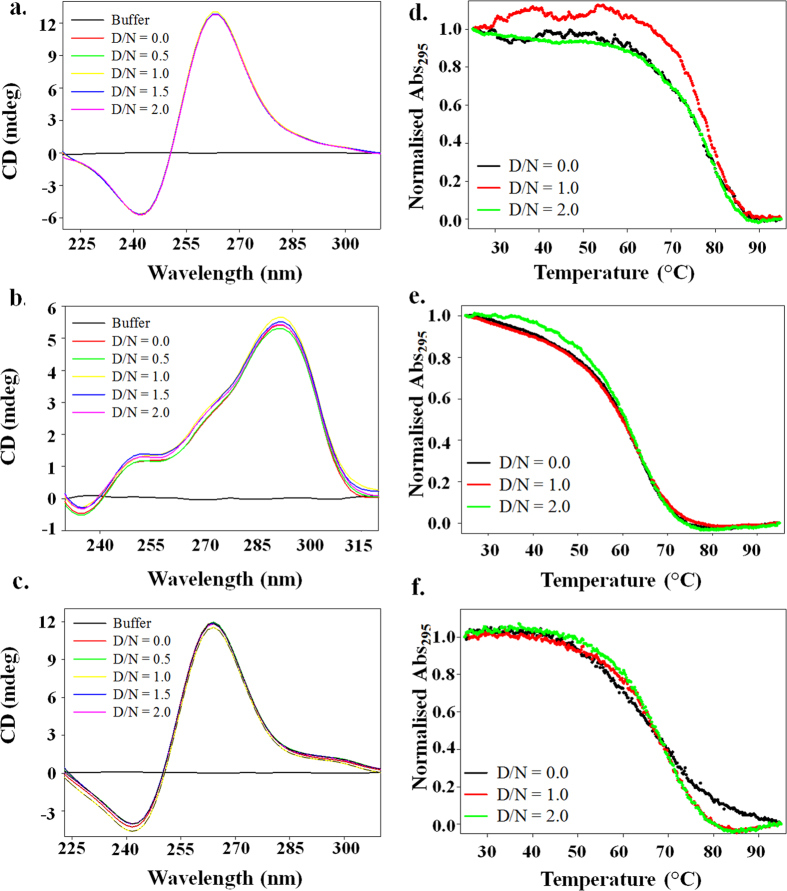
Circular Dichroism and thermal denaturation studies. Left Panel showing Circular Dichroism titration spectrum for free G-quadruplex DNA (Red) (**a**) Pu24T, (**b**) tel22 and (**c**) c-kit21 and in the presence of Piperine as a function of increasing concentration of Piperine upto D/N ratio = 2.0. D = Drug; N = Nucleic acid. Right panel showing thermal denaturation profile of (**d**) Pu24T G-quadruplex DNA, (**e**) tel22 G-quadruplex DNA and (**f**) c-kit21 G-quadruplex DNA in the absence and presence of Piperine upto D/N = 2.0.

We have performed circular dichroism (CD) titration experiment that monitors the changes in the secondary structure of DNA. As seen in Fig. 4a and c, the uncomplexed Pu24T and c-kit21 DNA displayed a positive peak around 260–265 nm and a negative peak at 240 nm, that are signature peaks for parallel G-quadruplex DNA topology^38^. While, a positive band at 280 nm with a hump at 255 nm and a negative peak at 240 nm is a characteristic of (3+1) hybrid topology of G-quadruplex, as showed by tel22 DNA^39^ (Fig. 4b). The perturbations in G-quadruplex topology caused due to addition of ligand may attributes to distortion of its structure^40^. In our study, we have found that upon addition of Piperine, even at D/N = 2.0 ratio, there were no appreciable change in the peaks of G-quadruplex DNA, which directs that globally DNA remains in G-quadruplex topology^41^. Thus, our CD results showed that binding of Piperine does not hamper the G-quadruplex structure formed by Pu24T, tel22 and ckit-21 DNA^41^ significantly and could indicate that it stabilizes their G-quadruplex structure.

To further explicit the stability of G-quadruplex structure on binding of Piperine, thermal melting studies of DNA in absence and presence of Piperine were performed. The thermal behaviour of G-quadruplex DNA in the presence of ligand provides details about the conformational changes in terms of its stability upon addition of ligand. The melting curves were recorded at a wavelength of 295 nm for all the three G-quadruplex DNA sequences upto D/N = 2.0 ratio (Fig. 4d,e,f). In the absence of Piperine, the melting temperature (T_m_) of Pu24T, tel22 and ckit21 were 75.0 °C^42^, 60.0 °C^43^ and 66.6 °C^44^ respectively (see Supplementary Table S2). After addition of Piperine, it has been observed that T_m_ of the Pu24T DNA increased to 78.0 °C and 79.0 °C at D/N = 1.0 and 2.0 respectively. However, not much significant results were observed for tel22 and ckit-21 DNA. It is well known that a better stabilization of quadruplex structure could be an indication from increased melting temperature after addition of ligand^45^. Thus, our data implies that Piperine stabilizes Pu24T G-quadruplex DNA structure. Also, it has been reported that regulation of c-myc gene is closely related to the stabilization of quadruplex structure formed at its promoter region^46^. Therefore, Piperine might exert its anti-cancer activity by stabilizing G-quadruplex structure and regulates its expression in cancer cells. However, the differences in ΔT_m_ for all the three G-quadruplex DNA may originate from the different DNA-binding affinity of Piperine and also reflected its sequence specific binding property.

Moreover, we have also examined the presence of induced CD signal that indicated strong interaction between DNA and ligand. Generally, the presence of negative induced CD signal implies close interaction involving most likely an overlap of π–π systems and indicates end-stacking^12^ between the ligand and G-quadruplex DNA^47^. We have observed a negative induced signal at ~364 nm (that is in the absorption range of Piperine) at 4 equivalents (see Supplementary Fig. S2). Thus, our results indicated that Piperine stabilizes Pu24T G-quadruplex DNA by binding via end stacking mode.

### NMR experiment

**Figure 5:**
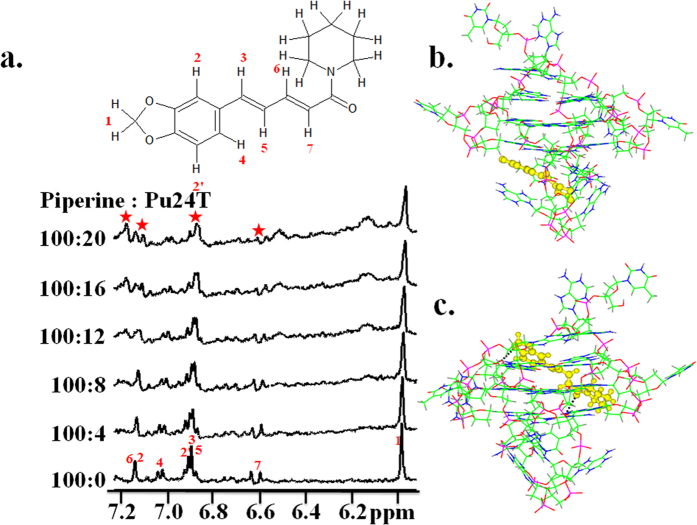
One dimensional proton spectra for Piperine - Pu24T complex and docking results. (**a**) NMR titration of 400 μM of Piperine with increasing concentration of Pu24T. (**b,c**) Both of the stable conformation of Piperine with Pu24T obtained from docking by Autodock 4.0 in which Piperine is shown in yellow color as ball stick representation. Black dotted lines showing hydrogen bonds between Piperine and Pu24T.

The chemical shift perturbations of ligand upon addition of DNA could provide the information about involvement of Piperine protons in its interaction with Pu24T DNA. For this, we have performed NMR titration experiment for Piperine and DNA. Upon titrating Pu24T DNA to Piperine solution, broadening and separation of Piperine proton resonances were observed (Fig. 5a). These protons mainly include resonances from conjugated system of Piperine like H7, H3, H5, H4 and H6. This could be possible only when Piperine molecule orient itself on Pu24T in such a way that its conjugated system and aromatic ring will come in contact with G-tetrads of Pu24T and stacks on it via π-π stacking.

**Figure 6:**
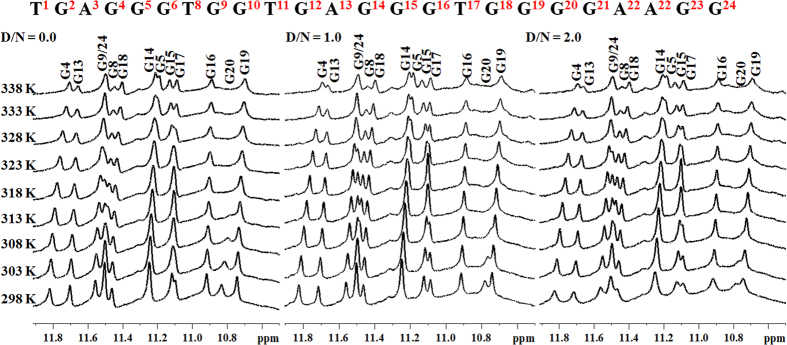
^1^H NMR spectra of Pu24T. ^1^H NMR spectra showing interaction of Piperine with Pu24T monitored by imino region as a function of temperature at ligand/DNA ratio = 0.0, 1.0 and 2.0.

**Figure 7:**
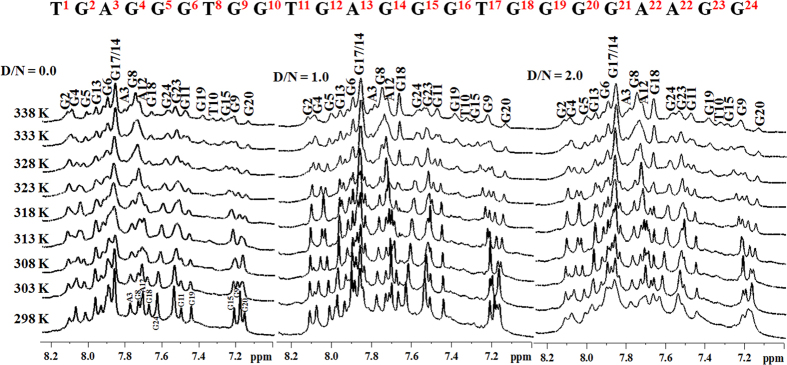
^1^H NMR spectra of Pu24T. ^1^H NMR spectra showing interaction of Piperine with Pu24T monitored by base proton region as a function of temperature at ligand/DNA ratio = 0.0, 1.0 and 2.0.

Moreover, we have also assessed the binding of Piperine to the Pu24T DNA using temperature-dependent NMR studies. The rise in temperature causes breaking of hydrogen bonds, due to which proton resonances of DNA become broadened and sometimes they may disappear. However, if ligand stabilizes the G-quadruplex structure, sharp peaks were observed at higher temperatures as compared to free DNA. In our study, with increase in temperature at D/N = 0.0, the imino proton resonances of bases like G13, G24, G8, G15 begin to broadens at 313 K (Fig. 6). The complete disappearance of resonances was not observed as melting temperature of Pu24T was higher than 338 K. However, at D/N = 1.0 and 2.0, the G8, G24 imino proton resonances can be seen as a sharp peaks upto 323 K and then slightly broadened above this temperature. It has also been observed that imino proton resonance of G15 base becomes sharp and clean at 323 K in D/N 1.0 and 2.0 which was otherwise broadened in D/N = 0.0 upto 333 K. All the above mentioned protons take part in the formation of upper and lower G-tetrad of Pu24T DNA. Additionally, resonance of G18 imino proton was also clearly seen upto 338 K after addition of Piperine in both the D/N ratios 1.0 and 2.0. Nevertheless, G18 base is a part of middle G-tetrad, but as seen from molecular dynamic simulation studies that Piperine molecule forms hydrogen bonds with G18 base (see Supplementary Fig. S6). This could account for the observed changes in G18 imino proton upon addition of Piperine. Also, in the base region of proton NMR spectra of Pu24T DNA, similar changes were observed. As depicted in Fig. 7, the resonances of guanine bases from upper and lower tetrad were sharp at higher temperature on addition of ligand as compared to D/N = 0.0. The resonances of G8, G6, G24 base protons were broadened at 308 K in D/N = 0.0, while at D/N = 1.0 and D/N = 2.0, these proton resonances were sharp upto 323 K. Likewise, G15H8 proton resonance was broadened at 308 K but it could be seen as separate peak upto 323 K in presence of Piperine. All these results clearly show that binding of Piperine stabilizes the G-quadruplex structure.

Further, we have also performed NMR titration experiment of Pu24T by incrementally adding Piperine to Pu24T DNA solution (see supplementary Fig. S3). The perturbations and broadening of DNA resonances were observed that indicated the formation of complex between Piperine and Pu24T, but, unfortunately, due to overlap of drugs and DNA resonances and aggregate formation of Piperine at very high concentrations, we could not get significant information from one-dimensional and two-dimensional NOESY experiments (see supplementary Fig. S4).

### Docking and Molecular Dynamics Simulation of *c-myc* G-quadruplex DNA – Piperine complex

**Figure 8:**
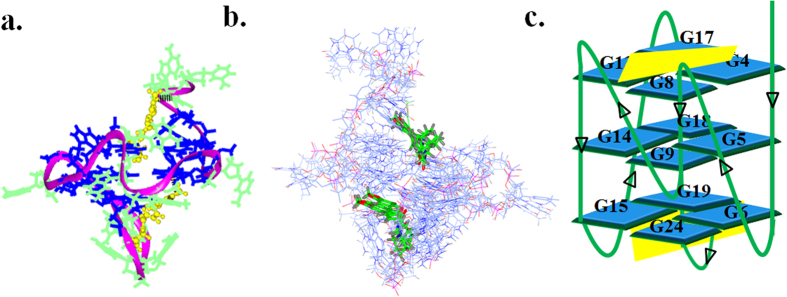
Energy minimized model of Piperine and Pu24T complex. (**a**) Lowest potential energy model of the complex after molecular dynamic simulation. Black dashes showing hydrogen bond formed between Piperine (yellow) and Pu24T (G-tetrads are shown in blue color). (**b**) Ensemble of ten lowest energy structures after molecular dynamics simulation (Piperine: yellow color, Pu24T: ice blue color). (**c**) Schematic representation showing Piperine (yellow) stacking at the top G-tetrad and below the bottom G-tetrad of Pu24T.

As from the results of above experimental data, it is clear that Piperine interacts with Pu24T with higher affinity; therefore, to get a better insight of this, we have performed molecular docking studies. The molecular structures of DNA as well as ligand were first optimized using Discovery Studio 3.5 (Accelrys Inc., USA). Docking was carried out by Autodock 4.0 using complete molecule of Pu24T in grid box. From docking result analysis, it was manifested that there were two separate sites on Pu24T for the possible binding of Piperine. Figure 5b and c shows the docked structures of Piperine with Pu24T DNA having the best binding energies at the two sites. It is noteworthy that the most potent binding site for Piperine is found to be located below the bottom G-tetrad having π-π interactions with G6 and binding energy of −7.18 kcal/mol (Site A) (see Supplementary Fig. S5a). As Piperine has a planar structure, it is expected that it could get stack at both the ends of the quadruplex^48^. Moreover, we have also found that second site is above the upper G-tetrad with but with weaker binding energy of −5.45 kcal/mol (Site B) (see Supplementary Fig. S5b). At this site also, the Piperine was stacked by π-π interactions with G17 base of Pu24T. It is also noteworthy that this G6 base and G17 bases are involved in the formation of upper and bottom G-tetrads of Pu24T G-quadruplex DNA. Further, we have also performed molecular dynamic (MD) simulation studies for the obtained docked structures on Discovery Studio 3.5. The 100 ns unrestrained MD simulations was performed and throughout the simulation it has been found that both the Piperine molecules remained bound to the Pu24T G-quadruplex DNA. Figure 8a shows a stable model for the Piperine - Pu24T complex obtained after simulation in which each molecule of Piperine is located at both the G-tetrads. In the lowest potential energy model, the O9 of one of the Piperine molecule was hydrogen bonded with H22 of G8 base of Pu24T. This G8 takes part in the formation of upper G-tetrad and thus Piperine molecule stably stacks at upper G-tetrad. Figure 8b shows the overlay of 10 lowest potential energy conformers obtained after unrestrained dynamic simulation.

Coalescing the experimental studies and computational simulation studies, it could be explained that Piperine binds to Pu24T at two sites that is at both the terminal G-tetrads and stabilizes the structure by formation of π–π interactions and hydrogen bonds (Fig. 8c).

**Figure 9:**
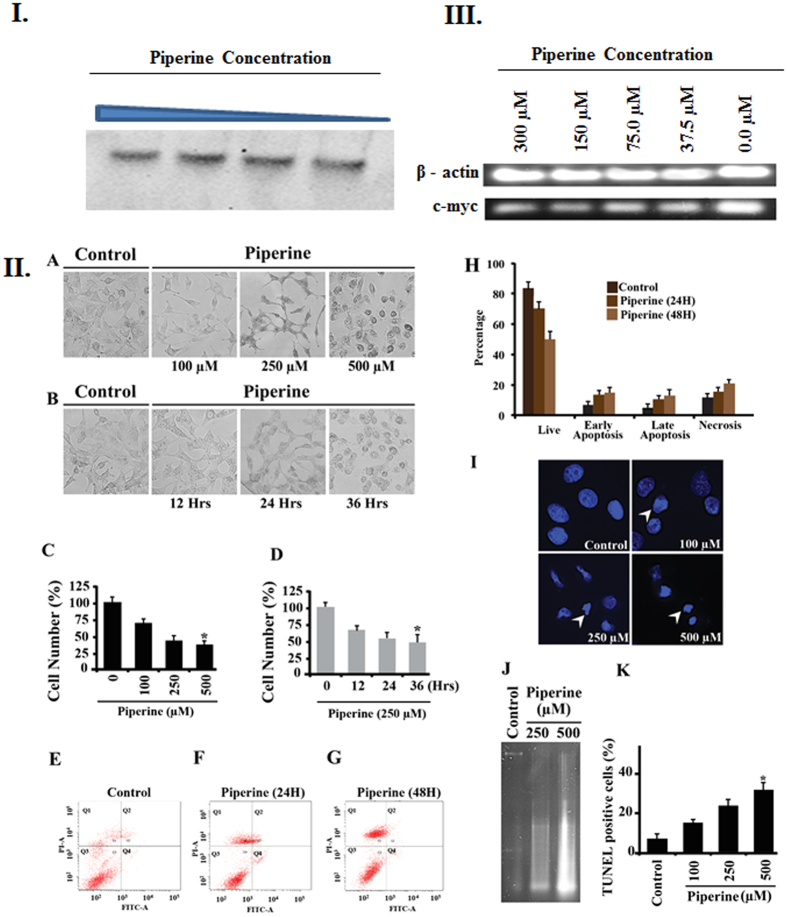
Gel mobility shift assay and evaluation of cytotoxicity of Piperine on cancer cells. 9I. Gel mobility shift assay. Increasing concentrations of Piperine was incubated with Pu24T. The shift in the mobility of DNA was assessed by running 20% Native poly acrylamide gel. The observed shift in DNA bands could be due to intercalation of Piperine to Pu24T DNA (Full-length gels image is available at Supplementary Information Fig. S9). **9II. Exposure to Piperine results in reduced cell viability and apoptosis in A549 cells.** (**A,B)** Cells were treated with Piperine in concentration (**A**) and time (**B**) dependent manner and bright field images were acquired. **(C,D)** Cell counting was done using bright field images of cells, treated with Piperine in concentration (**C**) and time (**D**) dependent manner. As shown in bar graphs (**H**) apoptosis was assessed in A549 cells, by flow cytometry analysis, using Annexin V-FITC and Propidium Iodide double staining. Cells were treated with 250 μM Piperine, for 24 hrs (**F)** and 48 hrs (**G)** DMSO was used as control **(E**,**I**) Nuclear morphology of Piperine treated cells was observed using DAPI staining. **(J,K)** DNA fragmentation was assessed in Piperine treated cells as shown in agarose gel (**J**) TUNEL assay (**K**) DNA fragmentation confirmation. **9III. Representative semi-quantitative RT-PCR analysis.** β-actin was used as internal control (Full-length gel image is available at Supplementary Information Fig. S10).

It is always a requirement to assess the cytotoxicity of drug in cancer cell lines and to deduce its mechanism of action. But, prior to this, we have performed Gel mobility Shift assay to confirm the binding of Piperine to Pu24T. Gel mobility shift assay was performed by incubating 20 μM of Pu24T with increasing concentration of Piperine for 1 hr at room temperature. With the increase in concentration of Piperine, there was shift (retardation) in the mobility of Pu24T DNA (Fig. 9I). The observed shift in DNA bands could be due to binding of Piperine to DNA and resulting in the formation of Pu24T-Piperine complex. Further, we have also observed that shift in mobility of DNA was maximum for complex formed between Pu24T DNA and Piperine followed by c-kit21 and then tel22 DNA (see Supplementary Fig. S9).

Moreover, we have also performed DNA Polymerase stop assay that confirms the stabilization of G-quadruplex structure formed by Pu24T DNA upon addition of Piperine. G-rich DNA templates hinder the activity of Taq Polymerase by formation of intramolecular G-quadruplex structures and this fact is being utilized in DNA polymerase stop assay. Ligands that stabilize G-quadruplex structure could lead to arrest of DNA synthesis process. The observed decreased in the intensity of PCR products with increasing concentration of Piperine indicates that Piperine stabilizes c-myc G-quadruplex DNA by blocking Taq Polymerase activity to amplify DNA(see Supplementary Fig. S7).

### Cells Exhibited Apoptotic Characteristics Following Piperine Treatment

In order to confirm the cytotoxicity of Piperine in cancer cells, we have treated A549 cells with Piperine. It has been observed that Piperine induces concentration (Fig. 9IIA) as well as time-dependent (Fig. 9IIB) apoptotic morphological changes in A549 cells. Cell counting data depicts reduced cell number, indicating anti-proliferative nature of molecule (Fig. 9IIC and IID). Flow cytometric analysis of Annexin V stained cells resulted in increased apoptotic fraction in Piperine treated cells, as compared to control (Fig. 9II E–H). As shown in Fig. 9I, apoptotic characteristics, such as nuclear shrinkage and fragmentation, were observed in Piperine treated cells, when stained with DAPI. Further, DNA fragmentation (Fig. 9IIJ), an important apoptotic feature was confirmed by agarose gel electrophoresis and TUNEL analysis (Fig. 9IIK). This reduced cell number and apoptotic characteristics such as cytoplasmic and nuclear condensation, externalization of membrane phospholipid phosphatidylserine and DNA cleavage, were observed from various experiments on exposure of cells to Piperine. In order to substantiate its cytotoxic effect on other cancer cell lines, we have performed MTT assay on HeLa, PC3, HepG2 and MCF-7 cell lines (see supplimentary Fig. S8a–d). We have found that Piperine shows concentration dependent cytotoxicity in all the cacerous cell lines used in this study that shows the potential of Piperine to inhibits various cancer cell growth. Furthermore, we have also employed semi - quantitative RT-PCR to understand the effect of Piperine on down-regulation of *c-myc* gene (Fig. 9III). This will allow us to semi-quantitate the expression of *c-myc* gene relative to a constitutively expressed housekeeping gene, β-actin. As shown in figure, a reduction in the level of *c-myc* mRNA in a dose-dependent manner (Fig. 9III) was observed and as it is clearly seen that β-actin mRNA is expressed likewise in both the control as well as in treated cells, thus, the reduction of mRNA level could be specific to *c-myc* gene.

## Conclusion

We have reported for the first time the binding of Piperine, a natural alkaloid, to various human G-quadruplex DNA sequences. We have found that Piperine has highest affinity for c-myc promoter region DNA sequence (Pu24T) forming G-quadruplex structure. The binding sites and its mode of binding on Pu24T were also determined. Further, its cytotoxic effect and mechanism of action on cancer cells lines was also evaluated. Together, our present observations in above studies gives us an idea of anti-proliferative and pro-apoptotic nature of Piperine and it exerts its anti-cancer activity could be by stabilizing the G-quadruplex structure formed at c-myc promoter region and down regulating its expression in cancer cells. This first report on the interactions of Piperine with G-quadruplex DNA would encourage the studies for molecular aspects of its anti-cancer mechanism emphasizing its potential to down-regulate c-myc gene expression.

## Methods

### Reagents and Cell lines

Piperine and other reagents used for buffer preparation such as NaCl, KCl, NaH_2_PO_4_, Na_2_HPO_4_, KH_2_PO_4_ and K_2_HPO_4_ (HPLC Grade) were purchased from Sigma Aldrich Chemicals Ltd. The solvents such as deuterium oxide, dimethyl sulphoxide (DMSO) were also procured from Sigma Aldrich Chemicals Ltd. All the reagents for PCR reaction like primers, dNTPs, *Taq* Polymerase was also obtained from Sigma Aldrich Chemicals Ltd.

Calf thymus DNA (CT-DNA) and G-quadruplex DNAs that is comprising of central guanine tracks of c-myc gene that is Pu24T-c-myc (d-5′-TGAGGGTGGTGAGGGTGGGGAAGG-3′), tel22 (d-5′-AGGGTTAGGGTTAGGGTTAGGG-3′), and c-kit21 (d-5′-CGGGCGGGCGCGAGGAGGGG-3′) were also procured from Sigma Aldrich Chemicals Ltd., USA. CT-DNA solution was prepared in the sodium phosphate buffer and its concentration was measured spectrophotometrically. For quadruplex formation, oligomers were dissolved in phosphate buffer (10 mM (K^+^), pH 7.0) with 50 mM KCl. The oligomer was annealed by heating at 90 °C for 5 mins, followed by overnight incubation at room temperature to allow gradual cooling. All the biophysical experiments were performed in the above mentioned buffer otherwise stated separately.

Human lung cancer cell lines (A549), human prostate cancer cell lines (PC3), human liver cancer cell line (HepG2), Human cervical cancer cell line (HeLa) and human breast cancer cell lines (MCF-7) were purchased from National Centre for Cell Science (NCCS), Pune, India. Growth media like Dulbecco’s modified Eagle’s medium (DMEM), Ham’s F12 medium, Minimum essential media Eagle (MEM) were purchased from Life Technologies (Gaithersburg, MD, USA). Cell culture reagents were purchased from Sigma. 4′, 6-Diamidino-2-phenylindole (DAPI) was obtained from Life Technologies. Phenol: Chloroform: Isoamyl alcohol mixture (25:24:1 v/v) was obtained from Himedia. DeadEnd fluorometric TUNEL system was obtained from Promega. FITC Annexin-V-Apoptosis Detection Kit I was purchased from BD Pharmingen. Cells-to-cDNAII Kit (Ambion) was purchased from Invitrogen.

### Fluorescence Titrations

Fluorescence titration experiment was performed on Synergy H1 multi-mode microplate reader using 96-well microplates at 25 °C. The excitation and emission wavelengths for Piperine were obtained by performing its absorption and fluorescence scan diluted in potassium phosphate buffer. The readings were taken for Piperine: G-quadruplex DNA titration at emission wavelength of 486 nm when excited at the wavelength of 341 nm. Each sample was tested in duplicates in 75 μL reaction volume. 5–10 μM of G-quadruplex DNA and a final concentration of 100 μM for CT-DNA were serially diluted; with the last well serve as blank (no DNA). Data were analyzed using SigmaPlot 12.0 software (Systat Software, Chicago, USA) according to the following equation that accounts for two receptor binding sites with two different affinities k_d_1 and k_d_2:


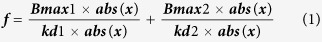


B_max_ = maximum number of binding sites.

K_d_ = equilibrium binding constant.

### Time-resolved fluorescence measurements

Time resolved fluorescence decays were collected on a Time-Correlated Single-Photon Counting (TCSPC) Spectrofluorometer (Horiba). A fixed wavelength Nano LED was used as the excitation source (k_ex_ = 375 nm), and emission was detected at a different wavelength. The fluorescence emission of Piperine and its complex with G-quadruplex DNA were counted with a micro channel plate photo multiplier tube after passing through the monochromator and were further processed through a constant fraction discriminator (CFD), a time-to-amplitude converter (TAC) and a multi-channel analyser (MCA). The fluorescence decay was obtained and further analysed using DAS software, provided by FluoroLog-TCSPC instruments.

### DNA Thermal denaturation experiments

DNA denaturation experiments were carried out on a Perkin Elmer Lambda 35 spectrophotometer equipped with Peltier temperature programmer (PTP 6+6) and water Peltier system PCB-1500. Melting curves for DNA were collected at a heating rate of 1 °C/min. in absence and presence of Piperine upto 2:1 Drug:DNA ratio. The normalized absorbance changes at 295 nm vs temperature were plotted.

### Circular Dichroism

The Circular Dichroism (CD) experiment was performed on a J-815 Spectropolarimeter (JASCO) equipped with peltier junction temperature controller. A quartz cuvette with 0.2 cm path length was used to record the spectra of samples containing 20 μM G-quadruplex and increasing concentrations of Piperine in 100 mM KCl, 10 mM phosphate buffer (K^+^) at pH 7.0. Spectra were recorded at 0.1 nm intervals from 200 nm to 350 nm with a 1 nm-slit width and averaged over three scans. Buffer CD spectra were subtracted from the CD spectra of DNA and the Drug-DNA complex.

### Nuclear Magnetic Resonance

NMR experiments were conducted on AVANCE 500 MHz BioSpin International AG, Switzerland equipped with a 5 mm broad band inverse probe. NMR studies were performed in H_2_O/D_2_O solvent at 9:1 ratio. For Piperine titration experiment, Pu24T was added to 400 μM Piperine solution upto D/N = 100:20 ratio and proton NMR spectra were collected at 298 K for each titration step. For DNA titration experiment, 3.85 mM of Pu24T-G-quadruplex DNA was prepared in 500 μL potassium phosphate buffer and final concentration of Piperine at D/N = 2.0 ratio was 7.7 mM. NMR data were processed, integrated and analysed on Topspin (1.3 version) software. NMR samples were referenced with 3 - (Trimethylsilyl) propionic-2, 2, 3, 3-d_4_ acid sodium salt (TSP).

### Docking and Molecular Dynamics simulation

The structure of G-quadruplex Pu24T (PDB code: 2MGN^49^) was taken as the starting model and the required replacements, addition of residues, optimization of G-quadruplex structure and Piperine structure using charmM forcefield were performed on Discovery studio 3.5 The molecular docking studies were carried out on Autodock 4.0 in which G-quadruplex DNA was treated as rigid body. All the other parameters used were set to their default values. Pu24T and Piperine structures were converted to AD4 format files and Gesteiger charges were assigned to the atoms. The grid was set in such a manner that it covers complete DNA structure so that ligand can explore the whole conformational space. The Lamarckian genetic algorithm^50^ was used for the search and the results were analyzed based on binding energy. For molecular dynamic (MD) simulation studies, the best conformation of Pu24T- Piperine complex obtained from docking studies was used as input. Second molecule of Piperine was placed manually in a way as obtained from docking studies. The complex was typed in charmM forcefield^51^ and solvated with periodic TIP3P^52^ orthorhombic water box containing 2091 water molecules. This complex was first minimized then subjected to simulated annealing molecular dynamics by employing standard dynamic cascade. In this cascade, the system was heated to 700 K and equilibrated for 10 ps under constant pressure. The production was done at 300 K for 100 ns in an NPT ensemble and long range electrostatics were treated with the Particle Mesh Ewald (PME) method^53^ with a 14 Å cut-off radius counted the non-bonded distances. The SHAKE algorithm^54^ was applied during the whole simulation runs in order to constrain the motion of Hydrogen bonds.

### Gel mobility shift assay

Gel mobility shift assay was performed by incubating 20 μM Pu24T and other G-quadruplex DNA with increasing concentrations of Piperine (0 to 30.0 mM) for 30 mins at room temperature and products were resolved on 20% NATIVE polyacrylamide gel [29:1 acrylamide/bis(acrylamide)] prepared by polymerizing acrylamide in 1X TBE containing phosphate buffer (10 mM (K^+^), 50 mM KCl). Gel was visualized by staining with ethidium bromide staining and analyzed on ImageQuant LAS 4000 (GE Healthcare).

### Cell Culture, DNA Fragmentation, TUNEL Assay, Morphological Evaluation and FACS Analysis of Apoptosis

A549 cells were cultured in Dulbecco’s modified Eagle’s medium (Life Technologies, Gaithersburg, MD, USA), supplemented with 10% fetal bovine serum, 100 μg/ml streptomycin and 100 U/ml penicillin at 37 °C with 5% CO_2_ under incubator. Cells were seeded in different types of tissue culture plates and at a confluency of 60–70%, they were used for various experiments. For morphological evaluation of apoptosis, bright field images were taken of cells treated with different concentrations and for various time periods with Piperine. Dimethyl sulfoxide (DMSO) treatment was used as control. Following cell counting, to assess cell viability, above described experimental cells were mounted with DAPI for nuclear morphological analysis. DNA fragmentation was observed in Piperine treated cells using agarose gel electrophoresis and TUNEL staining. DNA was isolated from Piperine treated cells using phenol-chloroform method and TUNEL staining was performed as per manufacturer’s instructions. For flow cytometric detection of apoptosis, cells were treated for different time periods with Piperine and stained with Annexin V-FITC and Propidium Iodide (PI), as per manufacturer’s instruction. FACS data was collected using BD FACSAria III Cell-Sorting System (BD, Bioscience) and analysis was done with FACS Diva software (Becton Dickinson, USA). The cytotoxic effects of Piperine was also examined on other 04 cancer cell lines by performing MTT (3-(4, 5-dimethylthiazol-2-yl)-2, 5 diphenyltetrazolium bromide dye) assay. For semi-quantitative RT PCR analysis, PC3 cells were grown in 6-well tissue culture plates and at 60–70% confluency, cells were incubated with various concentrations (300.0, 150.0, 75.0 and 37.5 μM) of Piperine for 24 h at 37 °C in humidified 5% CO_2_ incubator. Dimethyl sulfoxide (DMSO) treatment was used as control. Total RNA was prepared from treated and control cells and cDNA was prepared using Cells-to-cDNAII Kit (Ambion) according to the manufacturer’s protocol. Reverse transcriptase reaction was performed on Mastercycler Nexus Gradient (Eppendorf). The thermal cycling condition was programmed as 45 min at 45 °C, 10 min at 95 °C for one single cycle. Semi – quantitative PCR was performed using gene specific primers with the following sequences:

c-MYC (forward): 5′-CTTCTCTCCGTCCTCGGATTCT- 3′;

c-MYC (reverse): 5′-GAAGGTGATCCAGACTCTGACCTT-3′;

β-actin (forward): 5′- GAGCTACGAGCTGCCTGAC-3′;

β - actin (reverse): 5′-AGCACTGTGTTGGCGTACAG-3′.

## Additional Information

**How to cite this article**: Tawani, A. *et al*. Evidences for Piperine inhibiting cancer by targeting human G-quadruplex DNA sequences. *Sci. Rep.* **6**, 39239; doi: 10.1038/srep39239 (2016).

**Publisher's note:** Springer Nature remains neutral with regard to jurisdictional claims in published maps and institutional affiliations.

## Supplementary Material

Supplementary Information

## Figures and Tables

**Figure 1:**
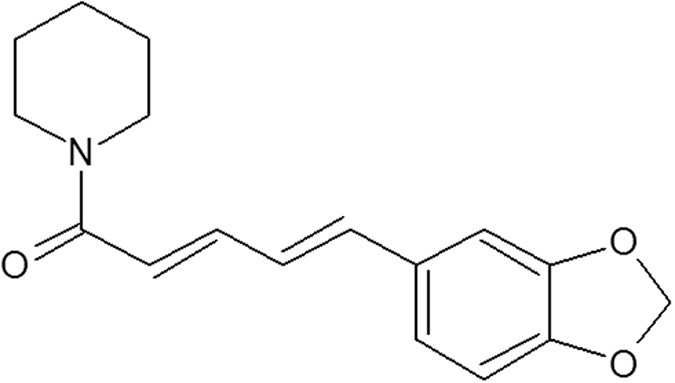
Structure of Piperine.

## References

[b1] Sen D. & Gilbert W. Formation of parallel four-stranded complexes by guanine-rich motifs in DNA and its implications for meiosis. Nature 334, 364–366 (1988). 3393228 10.1038/334364a0

[b2] Huppert J. L. & Balasubramanian S. Prevalence of quadruplexes in the human genome. Nucleic Acids Res. 33, 2908–2916 (2005). 15914667 10.1093/nar/gki609 PMC1140081

[b3] Wang Y. & Patel D. J. Solution structure of the human telomeric repeat d[AG3(T2AG3)3] G-tetraplex. Structure 1, 263–282 (1993). 8081740 10.1016/0969-2126(93)90015-9

[b4] Yang D. & Hurley L. H. Structure of the biologically relevant G-quadruplex in the c-MYC promoter. Nucleos. Nucleot. Nucl. 25, 951–968 (2006). 10.1080/15257770600809913 16901825

[b5] Zahler A. M., Williamson J. R., Cech T. R. & Prescott D. M. Inhibition of telomerase by G-quartet DNA structures. Nature 350, 718–720 (1991). 2023635 10.1038/350718a0

[b6] Shay J. W. & Wright W. E. Telomerase therapeutics for cancer: challenges and new directions. Nat. Rev. Drug. Discov. 5, 577–584 (2006). 16773071 10.1038/nrd2081

[b7] Postel E. H., Mango S. E. & Flint S. J. A nuclease-hypersensitive element of the human c-myc promoter interacts with a transcription initiation factor. Mol. Cell. Biol. 9, 5123–5133 (1989). 2601711 10.1128/mcb.9.11.5123-5133.1989 PMC363664

[b8] Wiman K. G. . Activation of a translocated c-myc gene: role of structural alterations in the upstream region. Proc. Natl. Acad. Sci.USA 81, 6798–6802 (1984). 6593728 10.1073/pnas.81.21.6798 PMC392019

[b9] Mathad R. I., Hatzakis E., Dai J. & Yang D. c-MYC promoter G-quadruplex formed at the 5′-end of NHE III1 element: insights into biological relevance and parallel-stranded G-quadruplex stability. Nucleic Acids Res. 39, 9023–9033 (2011). 21795379 10.1093/nar/gkr612 PMC3203601

[b10] Gonzalez V. & Hurley L. H. The c-MYC NHE III(1): function and regulation. Ann. Rev. Pharmacol. Toxicol. 50, 111–129 (2010). 19922264 10.1146/annurev.pharmtox.48.113006.094649

[b11] Siddiqui-Jain A., Grand C. L., Bearss D. J. & Hurley L. H. Direct evidence for a G-quadruplex in a promoter region and its targeting with a small molecule to repress c-MYC transcription. Proc. Natl. Acad. Sci.USA 99, 11593–11598 (2002). 12195017 10.1073/pnas.182256799 PMC129314

[b12] Pagano B. . State-of-the-art methodologies for the discovery and characterization of DNA G-quadruplex binders. Curr. Pharm. Des. 18, 1880–1899 (2012). 22376104 10.2174/138161212799958332

[b13] Wu P. . Stabilization of G-quadruplex DNA with platinum(II) Schiff base complexes: luminescent probe and down-regulation of c-myc oncogene expression. Chemistry 15, 13008–13021 (2009). 19876976 10.1002/chem.200901943

[b14] Ou T. M. . Stabilization of G-quadruplex DNA and down-regulation of oncogene c-myc by quindoline derivatives. J.Med.Chem. 50, 1465–1474 (2007). 17346034 10.1021/jm0610088

[b15] Tawani A. & Kumar A. Structural Insight into the interaction of Flavonoids with Human Telomeric Sequence. Sci. Rep. 5, 17574 (2015). 26627543 10.1038/srep17574 PMC4667226

[b16] Pavan Kumar Y. . Fluorescent Dansyl-Guanosine Conjugates that Bind c-MYC Promoter G-Quadruplex and Downregulate c-MYC Expression. Chembiochem 17, 388–393 (2016). 26708259 10.1002/cbic.201500631

[b17] Srinivasan K. Black pepper and its pungent principle-piperine: a review of diverse physiological effects. Critical reviews in food science and nutrition 47, 735–748 (2007). 17987447 10.1080/10408390601062054

[b18] Zarai Z., Boujelbene E., Ben Salem N., Gargouri Y. & Sayari A. Antioxidant and antimicrobial activities of various solvent extracts, piperine and piperic acid from Piper nigrum. LWT-Food Sci. Technol. 50, 634–641 (2013).

[b19] Li S. . Antidepressant-like effects of piperine and its derivative, antiepilepsirine. J. Asian Nat. Prod. Res. 9, 421–430 (2007). 17701559 10.1080/10286020500384302

[b20] Parmar V. S. . The International Journal of Plant Biochemistry and Molecular BiologyPhytochemistry of the genus Piper. Phytochemistry 46, 597–673 (1997).

[b21] Mittal R. & Gupta R. L. *In vitro* antioxidant activity of piperine. Methods Find. Exp. Clin. Pharmacol. 22, 271–274 (2000). 11031726 10.1358/mf.2000.22.5.796644

[b22] Shrivastava P. . Anti-apoptotic and anti-inflammatory effect of Piperine on 6-OHDA induced Parkinson’s rat model. J. Nutr. Biochem. 24, 680–687 (2013). 22819561 10.1016/j.jnutbio.2012.03.018

[b23] Wadhwa S., Singhal S. & Rawal S. Bioavailability Enhancement by Piperine: A Review, Asian J. Biomed. Pharm. Sci. 4, 1–8 (2014).

[b24] Zutshi R. K., Singh R., Zutshi U., Johri R. K. & Atal C. K. Influence of piperine on rifampicin blood levels in patients of pulmonary tuberculosis. J. Assoc. Physicians India 33, 223–224 (1985). 4044481

[b25] Johnson J. J. . Enhancing the bioavailability of resveratrol by combining it with piperine. Mol. Nutr. Food Res. 55, 1169–1176 (2011). 21714124 10.1002/mnfr.201100117 PMC3295233

[b26] Samuel M., Oliver S. V., Coetzee M. & Brooke B. D. The larvicidal effects of black pepper (Piper nigrum L.) and piperine against insecticide resistant and susceptible strains of Anopheles malaria vector mosquitoes. Parasit. Vectors 9, 238 (2016). 27117913 10.1186/s13071-016-1521-6 PMC4847181

[b27] Ferreira C. . Leishmanicidal effects of piperine, its derivatives, and analogues on Leishmania amazonensis. Phytochemistry 72, 2155–2164 (2011). 21885074 10.1016/j.phytochem.2011.08.006

[b28] Doucette C. D., Hilchie A. L., Liwski R. & Hoskin D. W. Piperine, a dietary phytochemical, inhibits angiogenesis. J. Nutr. Biochem. 24, 231–239 (2013). 22902327 10.1016/j.jnutbio.2012.05.009 PMC3524266

[b29] Pradeep C. R. & Kuttan G. Piperine is a potent inhibitor of nuclear factor-kappaB (NF-kappaB), c-Fos, CREB, ATF-2 and proinflammatory cytokine gene expression in B16F-10 melanoma cells. Int. Immunopharmacol. 4, 1795–1803 (2004). 15531295 10.1016/j.intimp.2004.08.005

[b30] Zsila F., Hazai E. & Sawyer L. Binding of the pepper alkaloid piperine to bovine beta-lactoglobulin: circular dichroism spectroscopy and molecular modeling study. Agric. Food Chem. 53, 10179–10185 (2005). 10.1021/jf051944g 16366712

[b31] Zsila F., Matsunaga H., Bikádi Z. & Haginaka J. Multiple ligand-binding properties of the lipocalin member chicken α1-acid glycoprotein studied by circular dichroism and electronic absorption spectroscopy: The essential role of the conserved tryptophan residue. B iochim. Biophys. Acta 1760, 1248–1273 (2006). 10.1016/j.bbagen.2006.04.006 16813999

[b32] Suresh D. V., Mahesha H. G., Rao A. G. & Srinivasan K. Binding of bioactive phytochemical piperine with human serum albumin: a spectrofluorometric study. Biopolymers 86, 265–275 (2007). 17407131 10.1002/bip.20735

[b33] Haris P., Mary V., Haridas M. & Sudarsanakumar C. Energetics, Thermodynamics, and Molecular Recognition of Piperine with DNA. J. Chem. Inf. Model. 55, 2644–2656 (2015). 26523930 10.1021/acs.jcim.5b00514

[b34] Balasubramanian S., Hurley L. H. & Neidle S. Targeting G-quadruplexes in gene promoters: a novel anticancer strategy? Nat. Rev. Drug Discov. 10, 261–275 (2011). 21455236 10.1038/nrd3428 PMC3119469

[b35] Monchaud D. & Teulade-Fichou M. P. A hitchhiker’s guide to G-quadruplex ligands. Org. Biomol. Chem. 6, 627–636 (2008). 18264563 10.1039/b714772b

[b36] Yang D. & Okamoto K. Structural insights into G-quadruplexes: towards new anticancer drugs. Future Med. Chem. 2, 619–646 (2010). 20563318 10.4155/fmc.09.172 PMC2886307

[b37] Burge S., Parkinson G. N., Hazel P., Todd A. K. & Neidle S. Quadruplex DNA: sequence, topology and structure. Nucleic Acids Res. 34, 5402–5415 (2006). 17012276 10.1093/nar/gkl655 PMC1636468

[b38] Chen X. . The development of a light-up red-emitting fluorescent probe based on a G-quadruplex specific cyanine dye. RSC Adv. 6, 70117–70123 (2016).

[b39] Ambrus A. . Human telomeric sequence forms a hybrid-type intramolecular G-quadruplex structure with mixed parallel/antiparallel strands in potassium solution. Nucleic Acids Res. 34, 2723–2735 (2006). 16714449 10.1093/nar/gkl348 PMC1464114

[b40] Zamiri B., Reddy K., Macgregor R. B. & Pearson C. E. TMPyP4 Porphyrin Distorts RNA G-quadruplex Structures of the Disease-associated r(GGGGCC)n Repeat of the C9orf72 Gene and Blocks Interaction of RNA-binding Proteins. J. Biol. Chem. 289, 4653–4659 (2014). 24371143 10.1074/jbc.C113.502336 PMC3931028

[b41] Ranjan N., Andreasen K. F., Kumar S., Hyde-Volpe D. & Arya D. P. Aminoglycoside binding to Oxytricha nova telomeric DNA. Biochemistry 49, 9891–9903 (2010). 20886815 10.1021/bi101517e PMC3641841

[b42] Islam M. M., Fujii S., Sato S., Okauchi T. & Takenaka S. A Selective G-Quadruplex DNA-Stabilizing Ligand Based on a Cyclic Naphthalene Diimide Derivative. Molecules (Basel, Switzerland) 20, 10963–10979 (2015). 10.3390/molecules200610963 PMC6272171 26076114

[b43] Phan A. T. & Mergny J. L. Human telomeric DNA: G-quadruplex, i-motif and Watson-Crick double helix. Nucleic Acids Res. 30, 4618–4625 (2002). 12409451 10.1093/nar/gkf597 PMC135813

[b44] Fernando H. . A conserved quadruplex motif located in a transcription activation site of the human c-kit oncogene. Biochemistry 45, 7854–7860 (2006). 16784237 10.1021/bi0601510 PMC2195898

[b45] Katsuda Y. . A Small Molecule That Represses Translation of G-Quadruplex-Containing mRNA. J. Am. Chem. Soc. 138, 9037–9040 (2016). 27410677 10.1021/jacs.6b04506

[b46] Siddiqui-Jain A., Grand C. L., Bearss D. J. & Hurley L. H. Direct evidence for a G-quadruplex in a promoter region and its targeting with a small molecule to repress c-MYC transcription. Proc. Natl. Acad. Sci.USA 99, 11593–11598 (2002). 12195017 10.1073/pnas.182256799 PMC129314

[b47] Sabharwal N. C. . Investigation of the interactions between Pt(II) and Pd(II) derivatives of 5,10,15,20-tetrakis (N-methyl-4-pyridyl) porphyrin and G-quadruplex DNA. J. Biol. Inorg. Chem. 21, 227–239 (2016). 26748794 10.1007/s00775-015-1325-8 PMC4801998

[b48] Haider S. M., Neidle S. & Parkinson G. N. A structural analysis of G-quadruplex/ligand interactions. Biochimie 93, 1239–1251 (2011). 21635933 10.1016/j.biochi.2011.05.012

[b49] Chung W. J., Heddi B., Hamon F., Teulade-Fichou M. P. & Phan A. T. Solution structure of a G-quadruplex bound to the bisquinolinium compound Phen-DC(3). Angew. Chem. Int. Ed. 53, 999–1002 (2014). 10.1002/anie.201308063 24356977

[b50] Morris G. M. . Automated docking using a Lamarckian genetic algorithm and an empirical binding free energy function. J. Comput. Chem. 19, 1639–1662 (1998).

[b51] Brooks B. R. . CHARMM: the biomolecular simulation program. J. Comput. Chem. 30, 1545–1614 (2009). 19444816 10.1002/jcc.21287 PMC2810661

[b52] Jorgensen W. L., Chandrasekhar J., Madura J. D., Impey R. W. & Klein M. L. Comparison of simple potential functions for simulating liquid water. J. Chem. Phys. 79, 926–935 (1983).

[b53] Darden T., York D. & Pedersen L. Particle mesh Ewald: An N⋅log(N) method for Ewald sums in large systems. J. Chem. Phys. 98, 10089–10092 (1993).

[b54] Ryckaert J.-P., Ciccotti G. & Berendsen H. J. C. Numerical integration of the cartesian equations of motion of a system with constraints: molecular dynamics of n-alkanes. J. Comput. Phys. 23, 327–341 (1977).

