# Supplementary material for: Evidences for Piperine inhibiting cancer by targeting human G-quadruplex DNA sequences
Source: Sci Rep. 2016 Dec 20;6:39239. doi: 10.1038/srep39239 (PMC5171706; doi:10.1038/srep39239)
Supplement: Supplementary Information [file srep39239-s1.pdf]

Supplementary Information

Evidences for Piperine inhibiting cancer by targeting human G-quadruplex DNA sequences

Arpita Tawani[1], Ayeman Amanullah[2], Amit Mishra[2] and Amit Kumar[1,*]

* Corresponding author

Email: amitk@iiti.ac.in (AK)

[1]*Center for Biosciences and Biomedical Engineering, Indian Institute of Technology Indore Khandwa Road, Simrol, Indore 453552, Madhya Pradesh, India*

[2]*Cellular and Molecular Neurobiology Unit, Indian Institute of Technology Jodhpur 342011, Rajasthan, India*

Index

SI. Supplementary Tables S3-S4

Table S1. The binding constant (K_d,(M)) values of Piperine with G- quadruplex DNA and CT-DNA. S3

Table S2. The melting temperature values of G-quadruplex DNA in absence and presence of Piperine. S3

SII. Supplementary Figures S4-S10

Figure S1. Time resolved fluorescence decay profile of 40.0 μM Piperine (Black) and its complex with CT-DNA at D/N ratio = 1.0 (Red) and D/N ratio = 2.0 (Green). S4

Figure S2. CD spectrum of Pu24T DNA and its complex with Piperin showing Indcued CD signals S5

Figure S3. One dimensional proton spectra for Piperine and Pu24T complex. S6

Figure S4. NOESYspectra for Piperine and Pu24T complex. S7

Figure S5. Representation of docked structure of complex obtained from docking. S8

Figure S6. Representation of hydrogen bond formation. S9

Figure S7. PCR stop assay for Pu24T and Piperine. S10

Figure S8. Cytotoxic effect of Piperine various cancer cell lines. S11

Figure S9. Full image of Gel mobility shift assay. S12

Figure S10. Full image of semi-quantitaive RT-PCR. S13

SI. Supplementary Tables

Table S1. The binding constant (K_d,(M)) values of Piperine with G-quadruplex DNA and CT-DNA.

**DNA**	**Binding Constant**	
	**K_d1(M)**	**K_d2 (M)**
Pu24T	2.50×10^{-9}	1.14×10^{-6}
Tel22	8.75×10^{-8}	14.8×10^{-6}
c-kit21	1.12×10^{-8}	12.73×10^{-6}
CT-DNA	24.12×10^{-6}	--

Table S2. The melting temperature values of G-quadruplex DNA in absence and presence of Piperine.

DNA	Melting Temperature (°C)		
	D/N = 0.0	D/N = 1.0	D/N = 2.0
Pu24T	75.0	78.0	79.0
Tel22	60.0	61.0	62.0
c-kit21	66.6	67.2	67.4

SII. Supplimentary Figures.

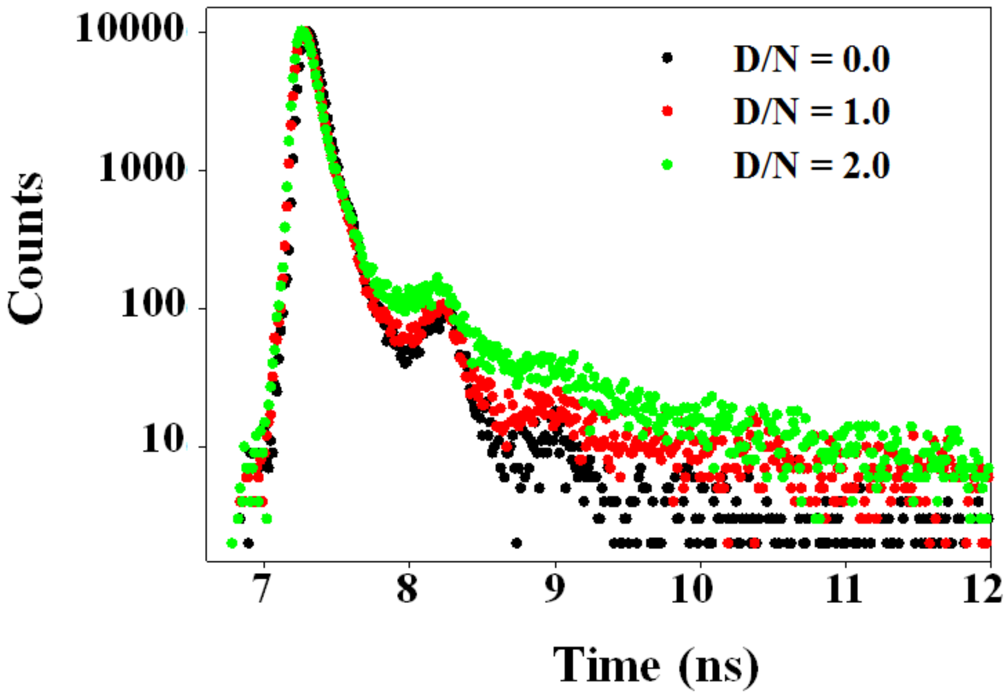

Figure S1. Time resolved fluorescence decay profile of 40.0 μM Piperine (Black) and its complex with CT-DNA at D/N ratio = 1.0 (Red) and D/N ratio = 2.0 (Green).

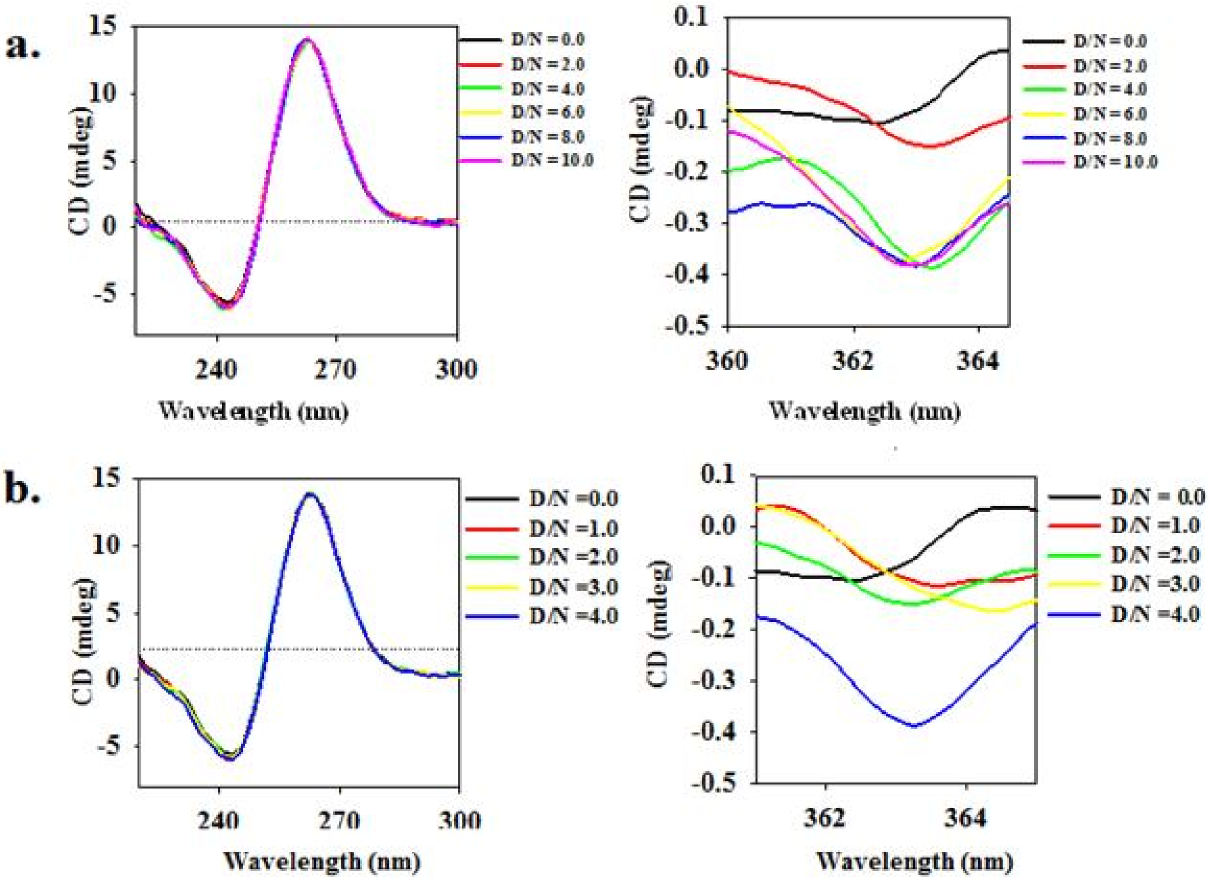

Figure S2. Circular Dichroism titration spectrum for free Pu24T (Black) and in the presence of Piperine as a function of increasing concentration of Piperine upto a. D/N ratio = 4.0, b. D/N ratio = 10.0. D= Piperine; N= Nucleic acid. Right hand images showing magnified ICD signals.

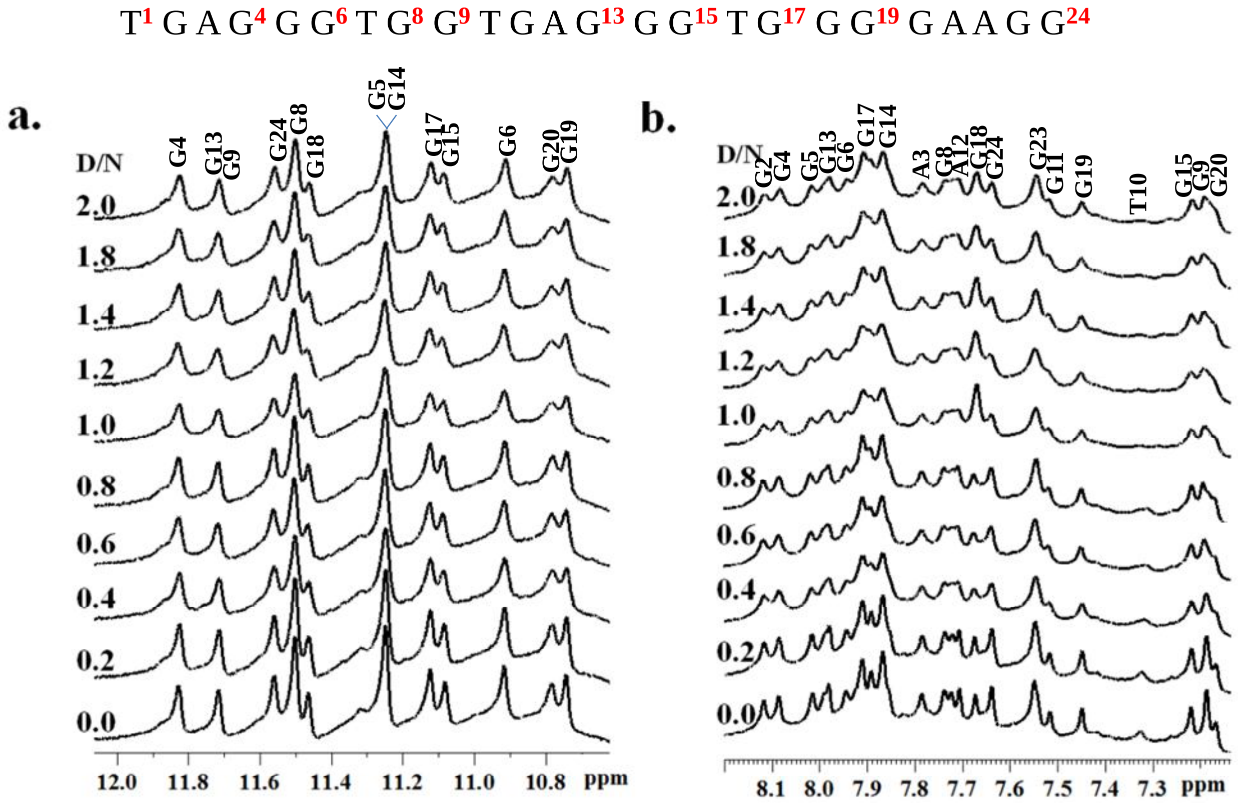

Figure S3. One dimensional proton spectra for Piperine and Pu24T complex. **a.** Imino region as a function of ligand/DNA (D/N) ratio at 298 K **b.** Base region as a function of ligand/DNA (D/N) ratio at 298 K.

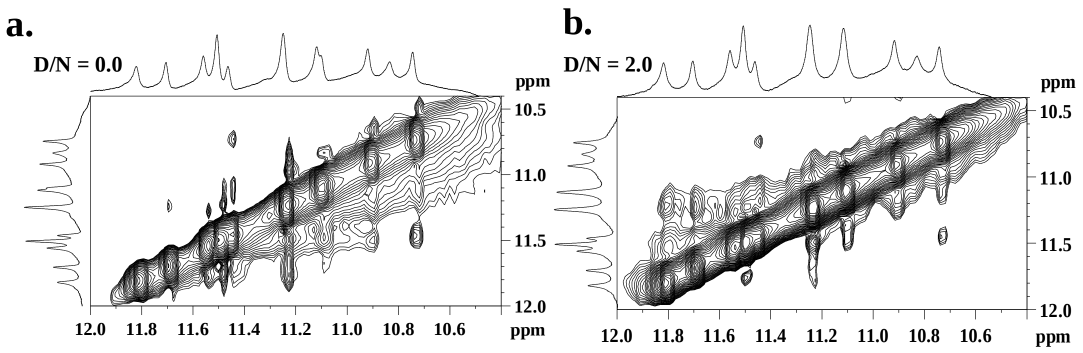

Figure S4a. Expansion of NOESY spectra showing imino region for Piperine and Pu24T-*c-myc* DNA as a function of ligand/DNA ratio A. D/N = 0.0 B. D/N = 2.0 at 298 K**.**

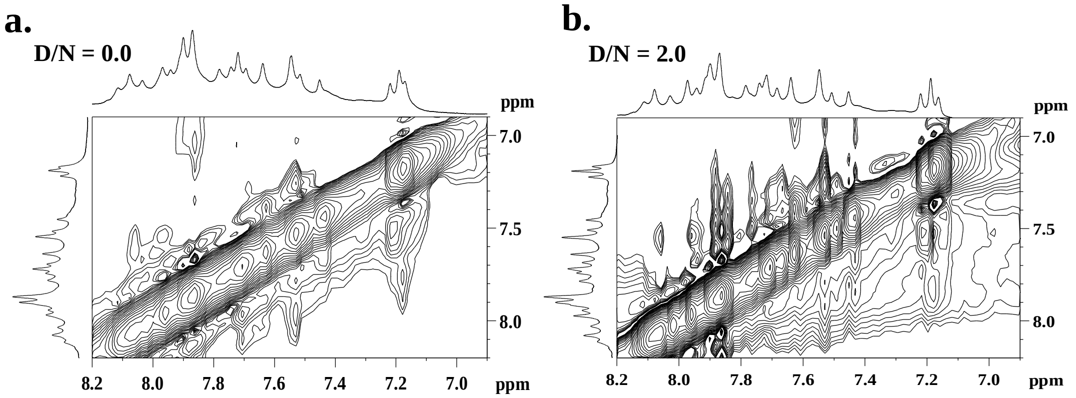

Figure S4b. Expansion of NOESY spectra showing base region for Piperine and Pu24T-*c-myc* DNA as a function of ligand/DNA ratio A. D/N = 0.0 B. D/N = 2.0 at 298 K.

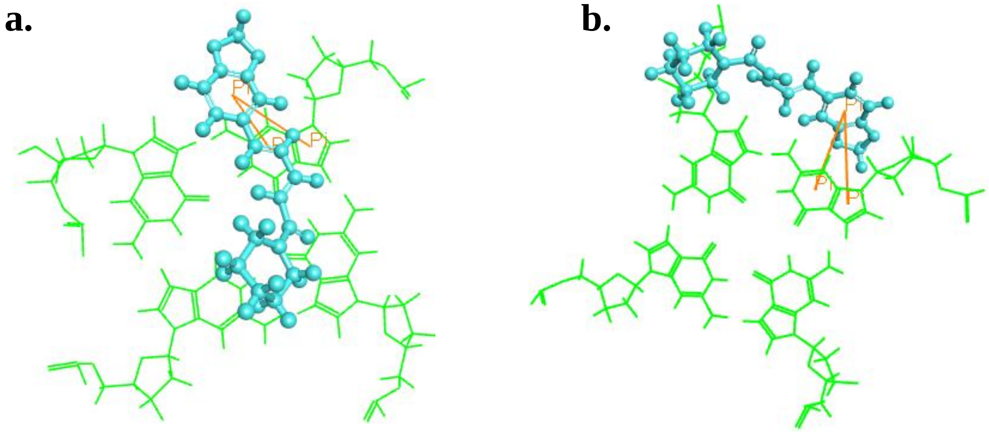

Figure S5. Representation of docked structure of complex obtained from docking by Autodock 4.0 showing π-π interactions (orange colored line) between Piperine (cyan) and G-tetrads (green) of Pu24T for both the sites.

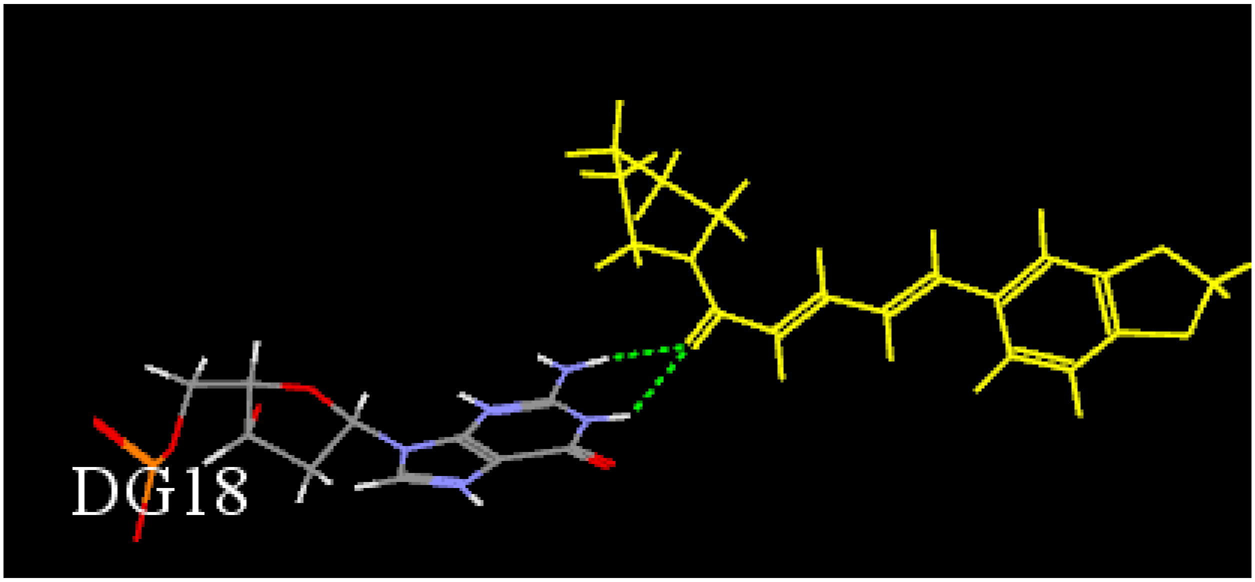

Figure S6. Representation of hydrogen bond (green) **formation** between G18 nucleotide and Piperine molecule (Yellow) as obtained from molecular dynamic simulation studies.

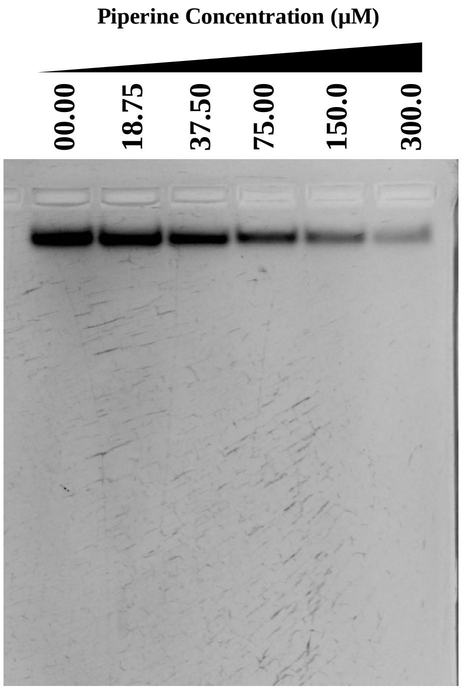

Figure S7. PCR stop assay with Pu24T: *Taq* Polynmerase stop assay has been employed by following a modified protocol of previous study using a test oligonucleotide Pu24T : d-(5'-TGAGGGTGGTGAGGGTGGGGAAGG-3') and a complementary oligonucleotide (RevPu24T): d-(5'-TTCTCGTCCTTCCCCA-3'). The observed decrease in the intensity of the PCR product with increasing concentration of Piperine indicates that binding of Piperine stabilizes the G-quadruplex strcuture formed by Pu24T DNA and blocks *Taq* polymerase activity. Amplified products were resolved on a 3% agarose gel in 1X TBE and stained with EtBr. Gel Image was analyzed on ImageQuant LAS 4000 (GE Healthcare).

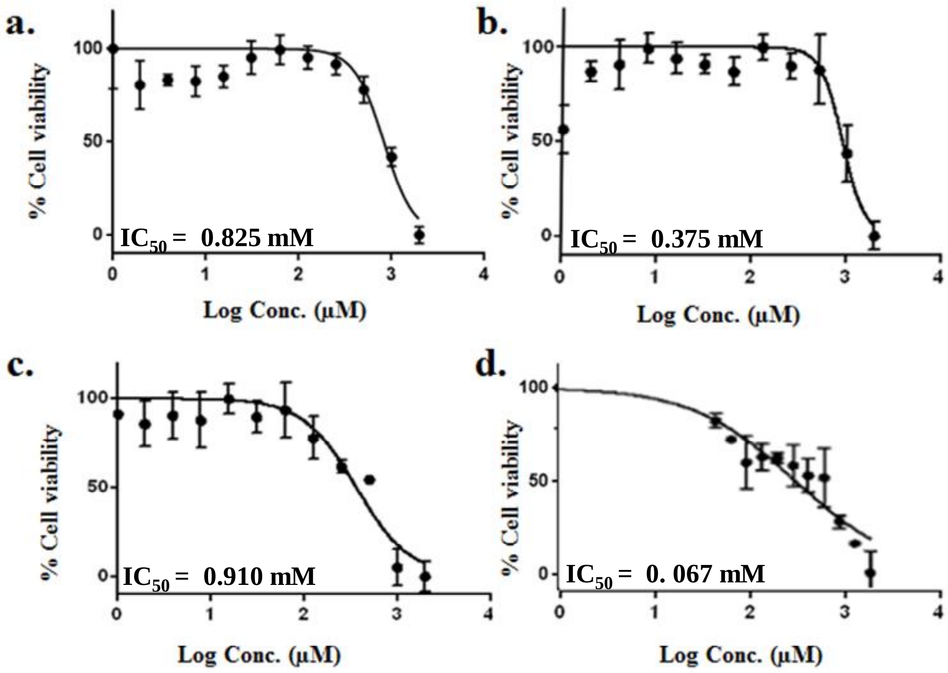

Figure S8. Cytotoxic effect of Piperine various cancer cell lines a. MCF-7 **b.** HepG2 **c.** HeLa **d.** PC3. IC_{50} values are mentioned at the bottom of plot**.**

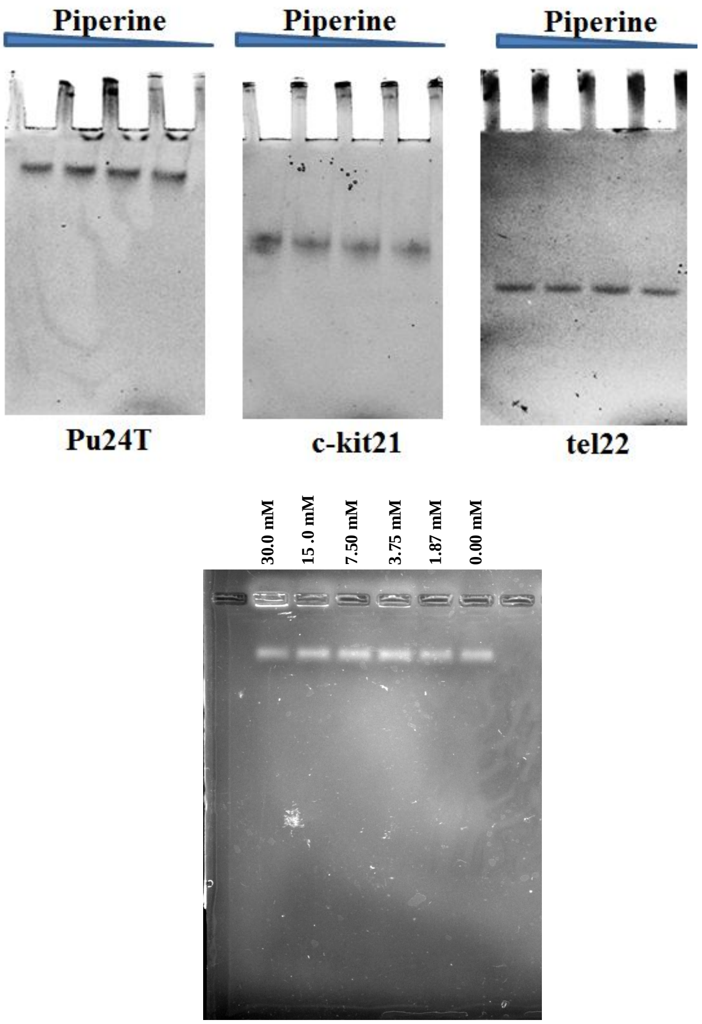

Figure S9. Full image of Gel mobility shift assay : Upper image : Native PAGE image for Increasing concentrations of Piperine incubated with various G-quadruplex DNA. Bottom image: Agarose gel image for increasing concentrations of Piperine (0, 1.88, 3.75, 7.50, 15.0 and 30.0 mM) was incubated with Pu24T DNA.

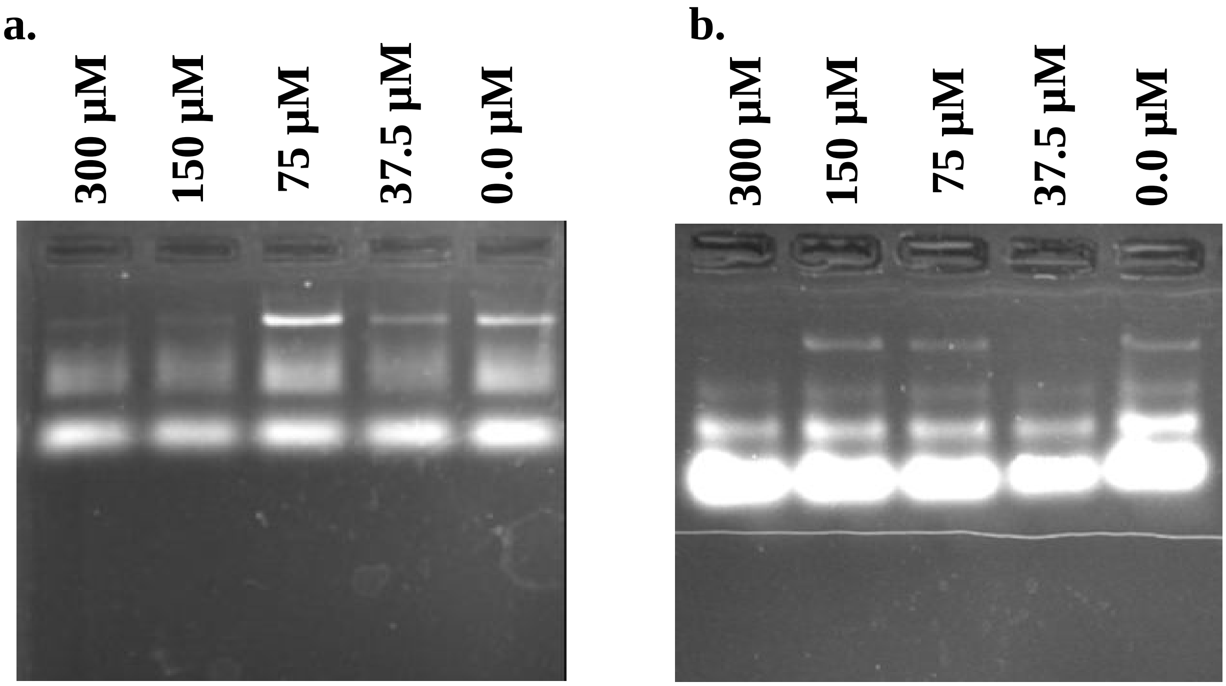

Figure S10. Full image of semi-quantitative RT-PCR analysis: a. c-myc b. β-actin as function of Piperine concentration.

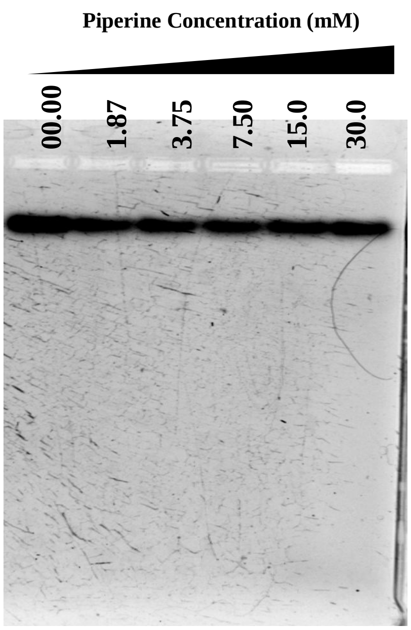

Figure S11. Gel mobility shift assay : Gel retardation assay for complimentary sequence of Pu24T that does not forms G-quadruplex structure with increasing concentration of Piperine. No significant shift in the mobility of DNA bands was observed.
